# Local-structure insight into the improved superconducting properties of Pb-substituted La(O, F)BiS_2_: a photoelectron holography study

**DOI:** 10.1038/s41598-025-86233-2

**Published:** 2025-03-11

**Authors:** YaJun Li, Yusuke Hashimoto, Noriyuki Kataoka, Zexu Sun, Sota Kawamura, Hiroto Tomita, Taro Setoguchi, Soichiro Takeuchi, Shunjo Koga, Kohei Yamagami, Yoshinori Kotani, Satoshi Demura, Kanako Noguchi, Hideaki Sakata, Tomohiro Matsushita, Takanori Wakita, Yuji Muraoka, Takayoshi Yokoya

**Affiliations:** 1 Engineering Research Center of Integrated Circuit Packaging and Testing, Ministry of Education, Tianshui Normal University, Tianshui, 741001 Gansu China; 2 Graduate School of Natural Science and Technology, Okayama University, Okayama, 700-8530 Japan; 3 Nara Institute of Science and Technology (NAIST), 8916-5 Takayama-cho, Ikoma, Nara, 630-0192 Japan; 4 Japan Synchrotron Radiation Research Institute (JASRI), 1-1-1 Kouto, Sayo-cho, Sayo-gun, Hyogo, 679-5198 Japan; 5 Department of Physics, College of Science and Technology(CST), Nihon University, 1-8-14, Kanda-Surugadai, Chiyoda-ku, Tokyo, 101-8308 Japan; 6 Tokyo University of Science, 1-3 Kagurazaka, Shinjuku-ku, Tokyo, 162-8601 Japan; 7 Research Institute for Interdisciplinary Science, Okayama University, Okayama, 700- 8530 Japan

**Keywords:** Physics, Condensed-matter physics, Superconducting properties and materials

## Abstract

Pb-substituted La(O, F)BiS_2_ (Pb-LaOFBiS_2_) exhibits improved superconducting properties and a resistivity anomaly around 100 K that is attributed to a structural transition. We have performed temperature(*T*)-dependent photoelectron holography (PEH) to study dopant incorporation sites and the local structure change across the anomaly. The PEH study of Pb-LaOFBiS_2_ provided evidence for the dominant incorporation sites of Pb and F: Pb atoms are incorporated into the Bi sites and F atoms are incorporated into the O site. No remarkable difference in the local structures around Pb and Bi atoms was observed. Across the temperature of the resistivity anomaly (T^*^), photoelectron holograms of Bi 4f changed. Comparisons of holograms with those of non-substituted LaOFBiS_2_ sample, as well as simulated holograms, suggested that (1), above T^*^, the tetragonal structure of Pb-LaOFBiS_2_ is different from the tetragonal structure of LaOFBiS_2_ and (2), below T^*^, the tetragonal structure still remains in Pb-LaOFBiS_2_. We discuss a possible origin of the difference in the structure above T^*^ and the implication of the result below T^*^, which are necessary ingredients to understand the physical properties of Pb-LaOFBiS_2_.

**Supplementary Information:**

The online version contains supplementary material available at 10.1038/s41598-025-86233-2.

## Introduction

**Fig. 1:**
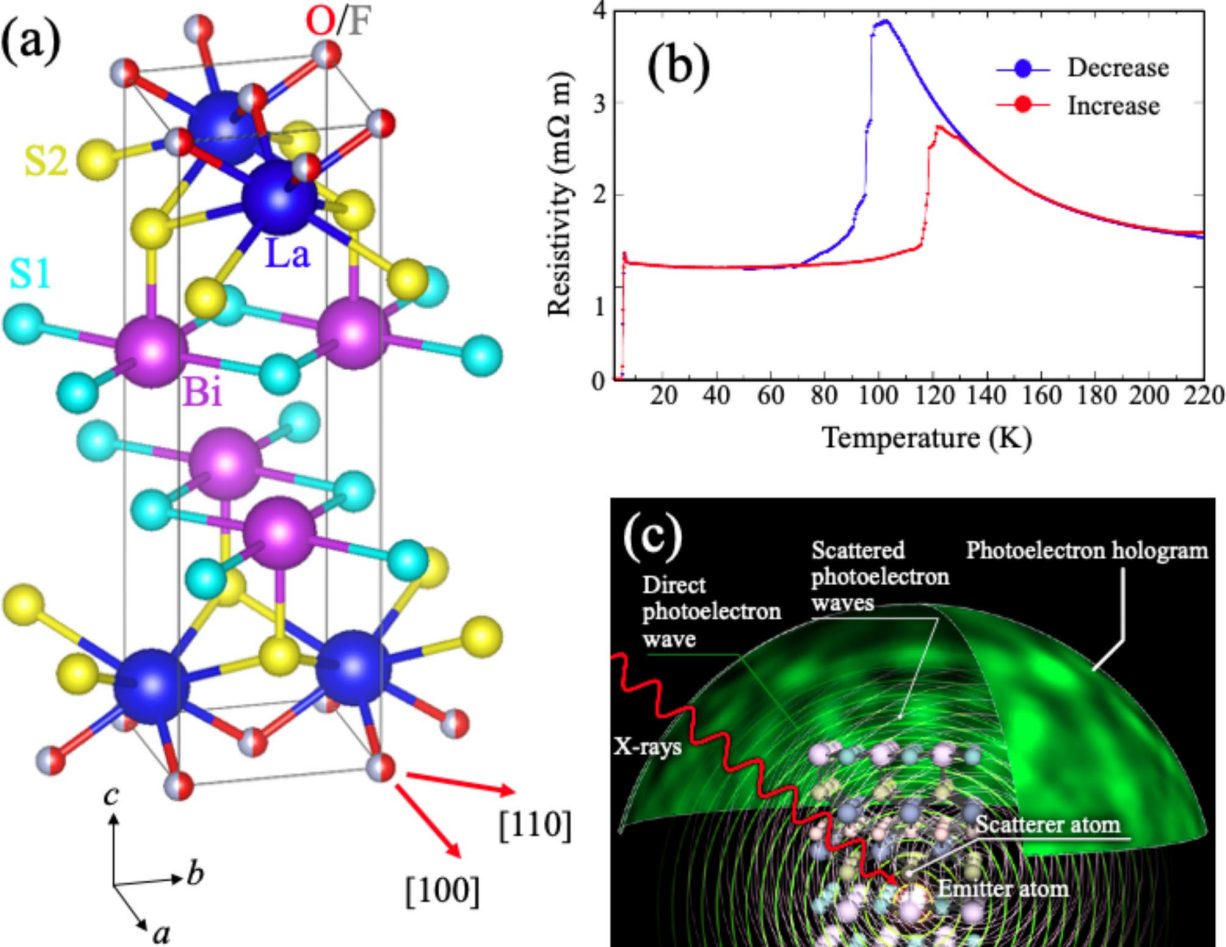
(**a**) Crystal structure of LaO_0.5_F_0.5_BiS_2_. VESTA^24^ was used to draw the crystal structures. (**b**) Temperature-dependent resistivity of LaO_0.5_F_0.5_Bi_1−*x*_Pb_*x*_S_2_ (*x* = 0.10). (**c**) Illustration of photoelectron holography. When soft X-rays are incident on a sample, an emitter atom will absorb the photon and emit a photoelectron as a spherical wave. A part of the photoelectron wave is scattered by the surrounding atoms (scatterers). The interference between the direct photoelectron wave and the scattered photoelectron waves forms a photoelectron hologram.

La(O, F)BiS_2_ is one family of the layered BiS_2_ superconductors, which consists of alternating stacks of a LaO blocking layer and a conducting BiS_2_ layer (Fig. 1(a))^1^. The superconductivity is induced by electron doping by substituting F for O, and the superconducting transition temperature (*T*_c_) changes with carrier concentration. The highest *T*_c_ of 10.6 K is obtained for La(O, F)BiS_2_prepared under high pressure^1,2^. Electron doping of LaOBiS_2_ by substitution of tetravalent Th^+4^, Hf^+4^, Zr^+4^ and Ti^+4^ for trivalent La^+3^has also been reported^3^. Moreover, RE (RE = La, Ce, Pr, Yb and Nd) site substitution in RE(O, F)BiS_2_ or in-plane S substitution with Se to introduce the chemical pressure also enhances the *T*_c_^4,5^. The crystal structure of the highest *T*_c_ La(O, F)BiS_2_ is not known because of the lower quality of samples synthesized by high pressure annealing. The crystal structure of high-pressure annealed La(O, F)BiS_2_, and its relation to the monoclinic La(O, F)BiS_2_ under pressure exhibiting *T*_c_of 10.7 K^6^, are important remaining questions.

Recently, Demura et al.. have succeeded in synthesizing Pb- or Sn-substituted RE(O, F)BiS_2_^7,8^. Both substitutions enhance *T*_c_ and also increase the superconducting volume fraction. Especially, in Sn-substituted La(O, F)BiS_2_, the highest *T*_c_ of 8.2 K was observed, although it was a filamentary superconductivity. Interestingly, both systems show a resistivity anomaly above *T*_c_, as shown in Fig. 1(b) for Pb-substituted La(O, F)BiS_2_, which has been attributed to a structural transition. More recently, an X-ray diffraction (XRD) study using single crystals of Sn-substituted La(O, F)BiS_2_revealed that the resistivity anomaly is due to a tetragonal to monoclinic structure transition^9^. The authors discussed a possible origin of the enhancement of *T*_c_ in terms of a mixture of tetragonal and monoclinic structure. For Pb-substituted La(O, F)BiS_2_, a XRD study using polycrystalline samples reported it to be a structural transition from a high-temperature tetragonal phase to a low-temperature lower symmetry phase^10^, although a definitive conclusion for the low-temperature phase has not been made. To understand the origin of the resistivity anomaly, and its relation to superconducting properties of Pb-substituted La(O, F)BiS_2_, further studies on the Pb doping site, Pb valence, and local structure change across the resistivity anomaly using other experimental techniques are necessary and valuable.

In this study, we used temperature(*T*)-dependent photoelectron holography (PEH) to study the local structure of Pb-substituted LaO_0.5_F_0.5_BiS_2_ and non-substituted LaO_0.5_F_0.5_BiS_2_. Hereafter, we call the former and the latter Pb-La(O, F)BiS_2_ and La(O, F)BiS_2_, respectively. PEH has the potential to determine the three-dimensional atomic structure around specific atoms in the crystal, and is especially useful to determine the incorporation sites of dopants^11,12^. We report experimental photo/Auger- electrons of La 4d, Bi 4f, Pb 4f, S 2p, O KLL and F KLL for Pb-La(O, F)BiS_2_, from which the occupation sites of the F and Pb atoms and possibility of the valence of Pb in Pb-substituted La(O, F)BiS_2_ will be discussed. Moreover, we observed the change in hologram pattern above and below the temperature of the resistivity anomaly (T^*^) in Pb-La(O, F)BiS_2_. From the comparison of the hologram patterns with those of La(O, F)BiS_2_ and the simulations, characteristics of the local structure of Pb-substituted La(O, F)BiS_2_ will be discussed.

**Fig. 2:**
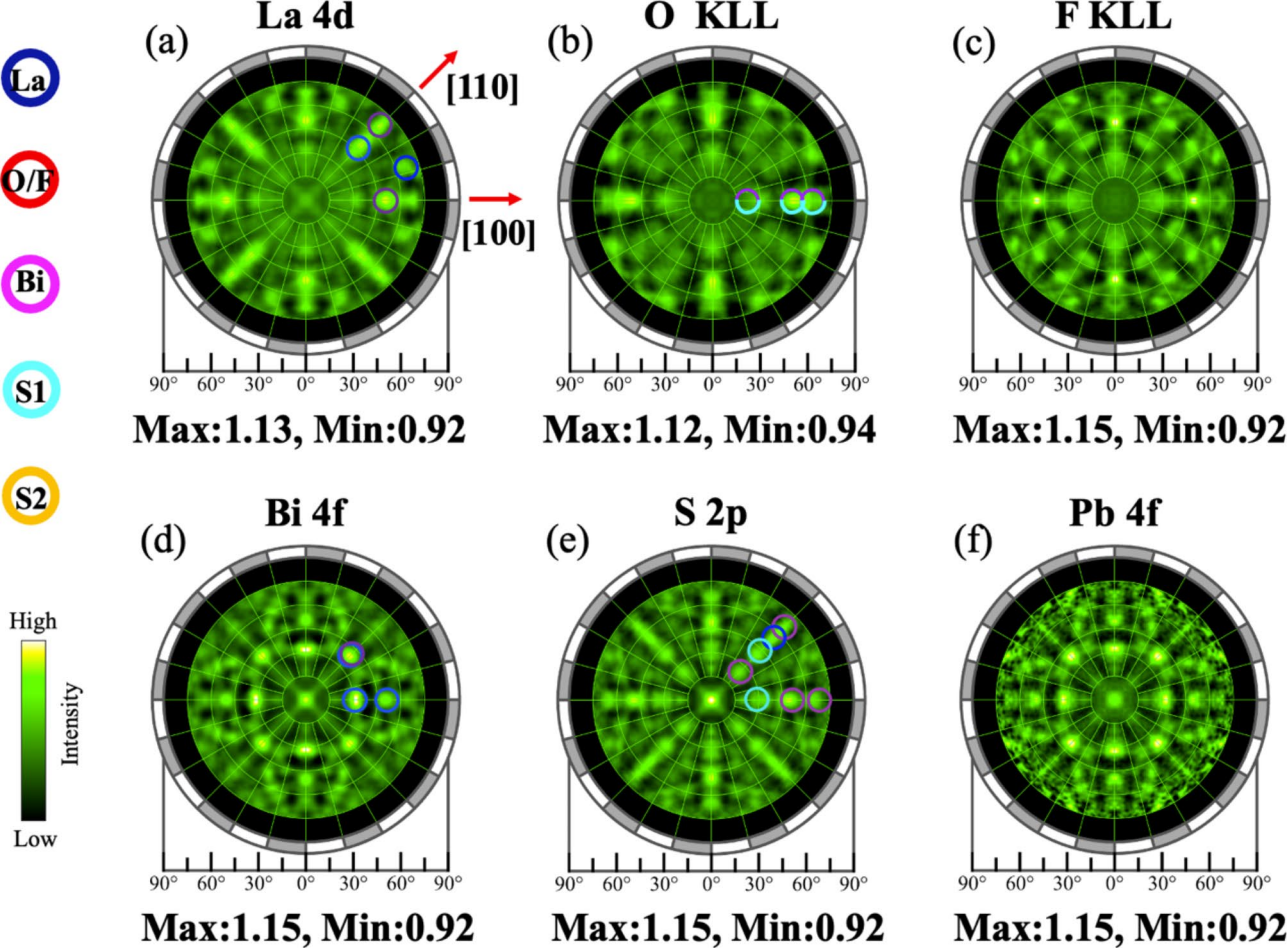
(**a–f**) Experimental holograms of La 4d, O KLL, F KLL, Bi 4f, S 2p and Pb 4f of Pb-La(O, F)BiS_2_, respectively. The maximum (Max) and minimum (Min) intensity values are indicated. The open circles in (**a**), (**b**), (**d**) and (**e**) are the expected FFP positions of neighboring scatterer atoms with respect to the emitter La, O, Bi, S atoms, respectively. The color of blue, red, purple, light blue, and yellow correspond to La O/F, Bi, S1(in-plane S), and S2 (out-of-plane S), respectively.

In the PEH technique, we measure the angular distribution of the core-level photoelectron or Auger-electron intensity, i.e., a photoelectron/Auger-electron hologram (Fig. 1(c)). Since the Bi 4f core level is composed of two structures^13^, we chose the kinetic energy for the higher kinetic energy component of the Bi 4f_7/2_peak to obtain a hologram having more bulk information. The holograms of the present study are characterized by bright spots named forward focusing peak (FFP), which correspond to the direction of a scatterer atom with respect to an emitter atom^12^. Figure 2(a) -(f) show photoelectron/Auger-electron holograms of La 4d, O KLL, F KLL, Bi 4f, S 2p and Pb 4f, respectively. The hologram of Pb 4f is smoothed to increase the signal-to-noise ratio (see Supplementary information for detailed description). In each hologram, we observe bright spots, some of which are superimposed by color open circles indicating the location of FFPs of scatterers (blue for La, red for O, purple for Bi, light blue for S1 at in-plane site, and yellow for S2 at out-of-plane site). For example, the hologram of La 4d shows two bright spots along the [100] and [110] directions and both are around the polar angle of approximately 50˚. The former is a FFP originating from the pairs of the emitter La and scatterer Bi atoms^13^, while the latter is a FFP originating from the pairs of the emitter La and scatterer La atoms. Since the hologram pattern reflects the local atomic arrangements around an emitter, the similarity of the hologram tells us the incorporation site of a dopant. In Fig. 2(b) and (c), the hologram of F KLL are similar to that of O KLL, indicating that F atoms are incorporated into the O sites, the same as for La(O, F)BiS_2_^13^, confirming that dominant F incorporation sites does not change with Pb substitution.

The hologram of Pb 4f looks similar to that of Bi 4f, as shown in Fig. 2(d) and (f). This indicates that the Pb atoms are predominantly incorporated into the Bi site, the same as Sn atoms in Sn-substituted La(O, F)BiS_2_^14^. For a more objective comparison, we calculated the squared error values between the smoothed holograms of Pb 4f and each of the holograms of La 4d, O KLL, F KLL, Bi 4f, and S 2p, using the standard deviation (see Supplementary information for detailed description). The values of relative standard deviations for La, O, F, Bi and S were 1.6, 1.6, 1.6, 1 and 1.4, respectively. When compared using the relative standard deviation of Bi as a reference, it is evident that the relative standard deviations of other images show significantly larger values. The smallest value of Bi 4f hologram supports the incorporation of Pb atoms into the Bi sites. The difference images of La 4d, O KLL, F KLL and S 2p, given in S1 of Supplementary information, show clear patterns that are similar to that of the Pd 4f hologram, In contrast, the difference image of Bi 4f does not show such a clear pattern. This suggests that the probability of Pb incorporation into other sites than Bi site is very small. The similarity of holograms between Pb and Bi also suggests that there is negligible distortion of the atomic arrangement around Pb atoms. Thus, the locations of Pb atoms in Pb-La(O, F)BiS_2_ are experimentally determined from the present study.

Since the valence state of Pb atoms in Pb-La(O, F)BiS_2_is important for understanding the carrier type, we discuss the valence of Pb atom. Unfortunately, we cannot evaluate the valence state of Pb in the sample by the chemical shift of the core level spectrum because reported core level binding energies do not show a clear dependence with respect to Pb valence^15^. To infer the valence of Pb in LaO_0.5_F_0.5_Bi_0.9_Pb_0.1_S_2_ sample, we associate the combination of Pb hologram and ionic radius (IR). The observation that the holograms of Bi 4f and Pb 4f are nearly identical suggests a negligible distortion of the atomic arrangement around Pb atoms. This indicates that the IR of Bi and Pb may be similar. Considering the effective ionic radius for the coordination number VI of Bi^3+^ (96 ~ 116 Å), Pb^2+^ (119 Å), and Pb^4+^(78 Å)^16^, we think that the valence of Pb is + 2 like, not + 4  like. This supports the speculation of Pb^2+^made in the previous report^8^, and is also in line with the dominant Pb^2+^core level observed in HXPES study^17^. As the valence of Bi ions is considered typically + 3  in La(O, F)BiS_2_, the + 2-like valence of the dopant Pb introduces hole carriers.

**Fig. 3:**
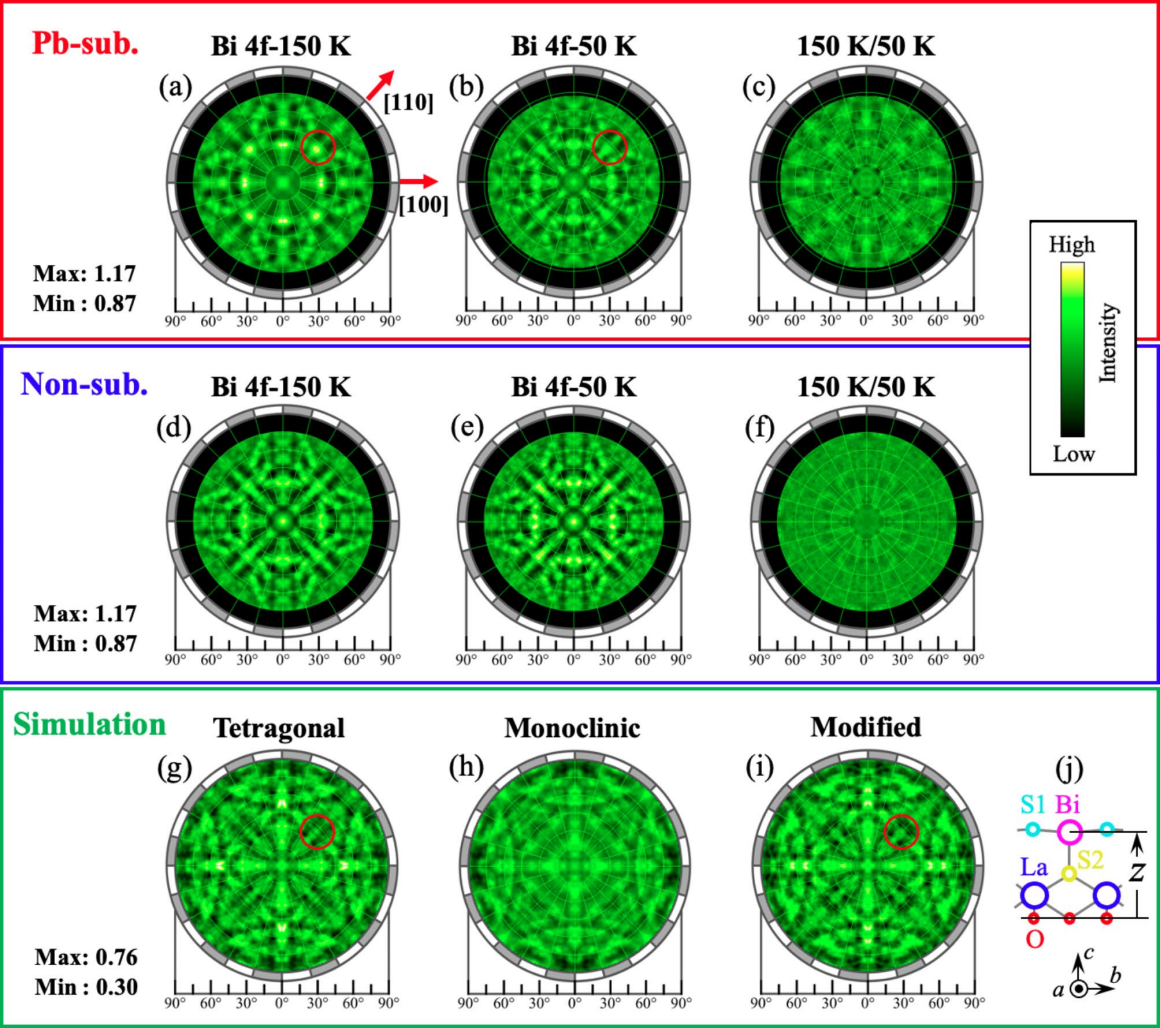
(**a-c**) Experimental Bi 4f holograms of Pb-La(O, F)BiS_2_ at 150 K, 50 K, and 150 K hologram divided by 50 K hologram, respectively. (**d-f**) the same as (**a-c**), but for La(O, F)BiS_2_. (**g**,** h**) simulated hologram based on a tetragonal structure model^18^, monoclinic structure model^6^, and the tetragonal structure mode with a Bi atom position shift by 0.2 Å. Maximum and minimum intensity values are indicated. The scale bar is for the experimental holograms.

As described in the introduction, Pb-La(O, F)BiS_2_ shows a resistivity anomaly that is attributed to a high-*T* tetragonal to low-*T* lower symmetry structural transition. We study the transition from a local structure point of view, by comparing Bi 4f holograms of Pb-La(O, F)BiS_2_ at 150 K and 50 K, with those of La(O, F)BiS_2_, as shown in Fig. 3(a)-(f). The holograms of La(O, F)BiS_2_measured at 150 K and 50 K represent those of the tetragonal crystal structure^18^. The holograms of Pb-La(O, F)BiS_2_ look slightly different between 50 K and 150 K. For example, intensities of the bright spot located along the [110] direction around 40° degree, which is indicated by a red circle in Fig. 3 (a) and (b), are higher at 150 K than at 50 K. The difference can be extracted by dividing the 150 K hologram with 50 K hologram, as shown in Fig. 3(c). In contrast, the holograms at 150 K and 50 K of La(O, F)BiS_2_ (Fig. 3(d) and (e)) show a similar pattern and consequently the division of holograms (Fig. 3(f)) shows a flat pattern, which is consistent with the absence of any anomaly between 50 and 150 K. These results indicate that the observed change of the holograms of Pb-La(O, F)BiS_2_ is related to the resistivity anomaly.

By comparing the holograms of Pb-La(O, F)BiS_2_ with that of La(O, F)BiS_2_ at the same temperature, we can examine the characteristic features of Pb-La(O, F)BiS_2_. Note that the hologram pattern of La(O, F)BiS_2_ represents that of the tetragonal structure. We can point out two observations. First, at 150 K, though the hologram pattern of the Pb-La(O, F)BiS_2_ is similar to that of La(O, F)BiS_2_, the intensity of the regions along the [110] direction around 40° relatively increases in Pb-La(O, F)BiS_2_, as indicated by the red circle in Fig. 3(a). Second, at 50 K, the hologram pattern of the Pb-La(O, F)BiS_2_ is similar to that of La(O, F)BiS_2_, though the amplitude of the pattern is smaller in Pb-La(O, F)BiS_2_ than in La(O, F)BiS_2_.

To consider the implication of these observations, we have simulated holograms for the tetragonal^18^and monoclinic^6^ structures, as shown in Fig. 3(g)-(i). The simulated hologram of the tetragonal structure model reproduces the hologram patterns at 50 K and 150 K of La(O, F)BiS_2_ reasonably well. The first observation indicates that the local structure around the Bi atoms of Pb-La(O, F)BiS_2_ is slightly different from that of La(O, F)BiS_2_, although both samples crystalize in the tetragonal structure. The intensity of the FFP of a Bi emitter and La or Bi scatterers, which is expected to appear in the region surrounded by the red circle in Fig. 3(a), was found to be reduced due to the defocusing effect in tetragonal La(O, F)BiS_2_^19^. Therefore, the change of the relative positions between the emitter Bi and scatterer Bi or La positions should occur, induced by the Pb substitution. Although we cannot examine all the possibilities, we found the change of the Bi *z* position (Fig. 3(j)) gives a noticeable effect on the intensity around the area of focus (the red circle in Fig. 3(g)). We show one simulated hologram in Fig. 3 (i), based on the tetragonal model with reduced Bi z value by 0.2 Å, which shows an increasing tendency of the intensity in the red circle.

Regarding the second observation, the surprising similarity of hologram pattern between Pb-La(O, F)BiS_2_ and La(O, F)BiS_2_ at 50 K suggests the existence of a tetragonal structure in Pb-La(O, F)BiS_2_ at 50 K. On the other hand, the reduced oscillation amplitude observed for Pb-La(O, F)BiS_2_ at 50 K suggests a mixture of another structure. The mean oscillation amplitude of the hologram of Pb-La(O, F)BiS_2_ is about half of that of La(O, F)BiS_2_, suggesting a roughly 50% inclusion of the tetragonal structure. A very recent study showed that a high-energy-resolution fluorescence detection mode for x-ray absorption near edge structure (HERFD-XANES) spectrum of Pb-La(O, F)BiS_2_ below T^*^is similar to a simulated spectrum based on the structure model for high-pressure(HP) phase^17^, which is a monoclinic structure. However, the smaller oscillation amplitude of the experimental HERFD-XANES spectrum than that of the simulated spectrum suggests that the HP structure is not the only structure below T^*^. In the present study, the reduced oscillation amplitude of the hologram observed for Pb-La(O, F)BiS_2_ at 50 K can be explained with a mixture of the tetragonal and monoclinic structures, as the hologram pattern of the monoclinic structure model has a weaker amplitude with a broader pattern. Thus, the present study is in line with the HERFD-XANES study. For Sn-La(O, F)BiS_2_, the recent XRD study reported a mixture of the tetragonal and monoclinic structures below T^*^ and discussed the relationship with the enhancing *T*_c_^9^. The observation that the large contribution of the tetragonal structure of Pb-La(O, F)BiS_2_ with enhancing superconducting properties may be related to the result of the structure study of Sn-La(O, F)BiS_2_. We hope that the present study motivates further studies on the origin of the enhancement of superconducting properties of Pb- and Sn-substituted La(O, F)BiS_2_.

In summary, the present PEH study provides experimental evidence that Pb atoms are incorporated into the Bi site and F atoms are incorporated into the O site. The very similar local structure between Bi and Pb, combined with the ionic radius, suggests that the valence of Pb is 2 + like, not 4 + like. Comparative *T*-dependent PEH studies of Pb-La(O, F)BiS_2_ and La(O, F)BiS_2_, combined with simulated results, showed (1) the difference in local structure of the tetragonal phase between Pb-La(O, F)BiS_2_ and La(O, F)BiS_2_ and (2) a marked contribution of the tetragonal phase in Pb-La(O, F)BiS_2_ at 50 K. This information is valuable to understand the impact of Pb substitution and its relation to the improved superconducting properties.

## Methods

### Sample synthesis and characterization

Single crystalline samples of LaO_0.5_F_0.5_Bi_0.9_Pb_0.1_S_2_ and LaO_0.5_F_0.5_BiS_2_are synthesized by a CsCl/KCl flux method in an evacuated quartz tube^8^. Powders of La_2_S_3_, Bi_2_O_3_, Bi_2_S_3_, PbF_2_ and BiF_3_ with Bi grains are used as starting materials. The Bi_2_S_3_ powders were prepared by reacting Bi and S grains in an evacuated quartz tube at 500 ˚C for 10 h. The mixture of starting materials and CsCl/KCl powder of 7.5 g were sealed in the evacuated quartz tube. The tube was heated at 900 ˚C for 12 h and kept at 900 ˚C for 24 h and cooled down to 630 ˚C at the rate of 0.5 ˚C/h or 1 ˚C/h.^8^ The starting nominal composition ratio of La : O : F : Bi : Pb : S was 1 : 0.62 : 0.38 : 0.90: 0.102. 00*l* peaks of X-ray-diffraction patterns for the crystal were indexed using the CeOBiS_2_-type structure with the space group *P*4/*nmm* symmetry. This indicated that the Pb substitution into La(O, F)BiS_2_ was successful. The Pb-La(O, F)BiS_2_ exhibits a first order transition around 110 K with a hysteresis of 30 K, as shown in Fig. 1(d).

### Photoelectron holography experiments

PEH measurements were performed at the beamline BL25SU at SPring-8 with the use of an upgraded retarding field analyzer (RFA)^20,21^, which is a home-made analyzer and has been applied for local-structure studies of various types of materials^14,22,23^. The resolving power *E/ΔE*and angular resolution of the RFA were set to 2000 and 0.5˚, respectively^21^. The photon energies of 900 eV for the measurement of Bi 4f and S 2p, 880 eV for the measurement of Pb 4f, and 845 eV for the measurement of La 4d were used, so that the kinetic energies of photoelectrons were around 740 eV. We also performed Auger electron holography for O KLL and F KLL. The sample temperatures were set to 50 K and 150 K, which are well below and above T^*^, respectively. All the measurements were carried out under a base pressure better than 5 × 10^−8^ Pa. Clean sample surfaces were obtained by cleaving the samples under vacuum just before the measurements.

### Simulations of holograms

Simulated holograms for comparison with the experimental holograms were created using the total analysis multiple scattering pattern simulation (TMSP) code^12^with an individual spherical-like cluster, in which an emitter atom was placed at the center. In the present study, to consider the effect of crystal termination at a surface, spherical dome-shaped atomic clusters with a radius of 2 nm were used; the distance between the atom at the center (emitter) and the surface depended on the position of the emitter^19^. Simulations were performed for an electron kinetic energy of 740 eV and a temperature of 150 K using an inelastic mean free path of 1.5 nm and a Debye temperature of 224 K^19^ for each atom. The obtained holograms for every emitter atom within a depth of 1.7 nm from the surface were summed, taking the mean free path into consideration, to obtain a final simulated hologram of an element.

## Electronic supplementary material

Below is the link to the electronic supplementary material.

Supplementary Material 1

## Data Availability

All relevant data are available from the corresponding authors upon reasonable request.
