# Supplementary material for: Local-structure insight into the improved superconducting properties of Pb-substituted La(O, F)BiS2: a photoelectron holography study
Source: Sci Rep. 2025 Mar 11;15:8366. doi: 10.1038/s41598-025-86233-2 (PMC11897217; doi:10.1038/s41598-025-86233-2)
Supplement: Supplementary file 1 — Supplementary Material 1 [file 41598_2025_86233_MOESM1_ESM.docx]

**Supplementary Information for “Local-structure insight into the improved superconducting properties of Pb-substituted La(O,F)BiS_2_: A photoelectron holography study”**

YaJun Li^1,2*^, Yusuke Hashimoto^3^, Noriyuki Kataoka^2^, ZeXu Sun^3^, Sota Kawamura^3^, Hiroto Tomita^3^, Taro Setoguchi^2^, Soichiro Takeuchi^3^, Shunjo Koga^3^, Kohei Yamagami^4^, Yoshinori Kotani^4^, Satoshi Demura^5^, Kanako Noguchi^6^, Hideaki Sakata^6^, Tomohiro Matsushita^3^, Takanori Wakita^2,7^, Yuji Muraoka^2,7^ and Takayoshi Yokoya^2,7*^

^1^Engineering Research Center of Integrated Circuit Packaging and Testing, Ministry of Education, Tianshui Normal University, Tianshui, Gansu 741001, China

^2^Graduate School of Natural Science and Technology, Okayama University, Okayama 700-8530, Japan

^3^Nara Institute of Science and Technology (NAIST), 8916-5 Takayama-cho, Ikoma, Nara 630-0192, Japan

^4^Japan Synchrotron Radiation Research Institute (JASRI), 1-1-1 Kouto, Sayo-cho, Sayo-gun, Hyogo 679-5198, Japan

^5^Nihon University, 4-8-24 Kudan-Minami, Chiyoda-ku, Tokyo102-8275, Japan

^6^Tokyo University of Science, 1-3 Kagurazaka, Shinjuku-ku, Tokyo 162-8601, Japan

^7^Research Institute for Interdisciplinary Science, Okayama University, Okayama 700-8530, Japan

*xiaotaiyang@tsnu.edu.cn; yokoya@cc.okayama-u.ac.jp

1. **Relative standard deviation for similarity evaluation and difference images**

We obtained the difference images ($\Delta\chi$) from the holograms (*χ*) using the following equations:

$${\Delta\chi}_{M}=\chi_{\mathrm{Pb}}-a\chi_{M}-b,$$

$$E_{M}=\frac{1}{N}\left\| {\Delta\chi}_{M} \right\|^{2} ,$$

where *M* represents La 4d, O KLL, F KLL Bi 4f or S 2p. *N* is the number of data points of a hologram. *a* and *b* are constants used to normalize the intensities of the other holograms to be compared, taking a constant background into consideration, and are determined by

$$\frac{\partial E_{M}}{\partial a}=0, \frac{\partial E_{M}}{\partial b}=0.$$

We deﬁned the relative root mean square error values as

$\sigma_{M}=\frac{\sqrt{E_{M}}}{\sqrt{E_{Bi}}}$,

and obtained 1.6, 1.6, 1.6, 1 and 1.4 for La 4d, O KLL, F KLL, Bi 4f and S 2p, respectively.

The obtained difference images are shown in S1.


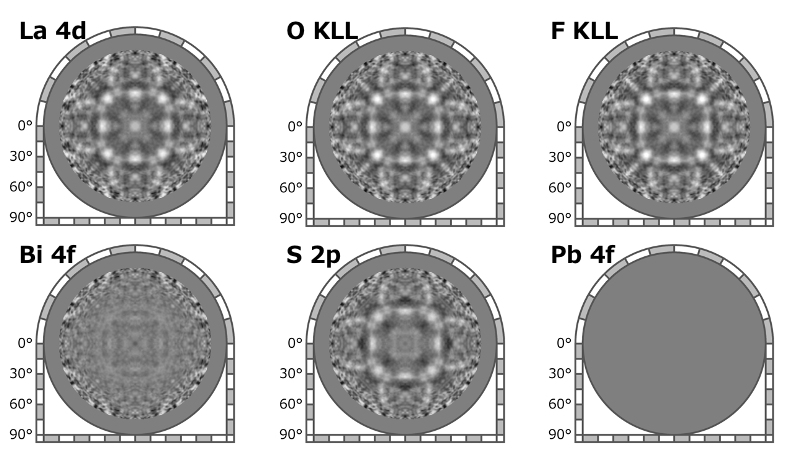


S1. Difference images of La 4d, O KLL, F KLL, Bi 4f, S 2p and Pb 4f. The same maximum and minimum values of the intensity window are used.

1. **Signal-to-noise (S/N) ratio**

For the comparison to the signal-to-noise(S/N) ratio, we have made a rough estimation of the S/N ratio of the smoothed Pb 4f hologram and other holograms using

$$\chi_{noise}=\chi-\chi_{smooth} ,$$

$${S/N}=\frac{\sigma_{smooth}}{\sigma_{noise}} ,$$

where $\chi_{smooth}$ is the smoothed $\chi$, and $\sigma_{smooth}$ and $\sigma_{noise}$ are standard deviations of $\chi_{smooth}$ and $\chi_{noise}$, respectively.

The estimated S/N ratio of the La 4d, O KLL, F KLL, Bi 4f, S 2p and smoothed Pb 4f holograms are 21, 21, 14, 16, 14 and 4, respectively. The S/N ratio of the smoothed Pb 4f hologram is relatively smaller than that of other holograms. However, the lower S/N ratio of the Pb 4f just increases the standard deviations in the similarity evaluation by the same constant, and therefore it does not affect the evaluation in principle. The variation of S/N ratios of the La 4d, O KLL, F KLL, Bi 4f and S 2p does not affect the evaluation because the error bars estimated as the inverse of the S/N ratio are at most +/- 7%, which is smaller than the difference of the standard deviations in the similarity evaluation.
